# Steam Oxidation of Austenitic Heat-Resistant Steels TP347H and TP347HFG at 650–800 °C

**DOI:** 10.3390/ma12040577

**Published:** 2019-02-14

**Authors:** Zhiyuan Liang, Qinxin Zhao

**Affiliations:** Key Laboratory of Thermal Fluid Science and Engineering of MOE, School of Energy and Power Engineering, Xi’an Jiaotong University, Xi’an 710049, China

**Keywords:** steam oxidation, grain size, heat-resistant steel, oxide scales

## Abstract

Steam oxidation of austenitic heat-resistant steels TP347H and TP347HFG at 650–800 °C was investigated. Comprehensive micro-characterization technologies containing Scanning Electron Microscope (SEM), Energy Dispersive X-ray Spectroscopy (EDS), X-ray Diffraction (XRD), and X-ray Photoelectron Spectroscopy (XPS) were employed to observe and analyze the oxidation products. Results show that breakaway oxidation behaviors were observed on TP347H at 700 °C and 800 °C. The oxidation kinetics of TP347HFG at 650–800 °C followed a parabolic law. The oxide scales formed on TP347HFG were composed of MnCr_2_O_4_ and Cr_2_O_3_. A thin and protective Cr-rich oxide scale was replaced by Fe_2_O_3_ nodules due to the insufficient outward migration of metallic ions, including Cr and Mn at the subsurface of coarse-grain TP347H. Smaller grain of TP347HFG promoted the formation of the compact Cr-rich oxide scales. At higher temperatures, the incubation period for breakaway oxidation of the Cr-rich oxide scale was much shorter because of quick evaporation of the Cr_2_O_3_ oxide scale and the slower outward diffusion of metallic ions via the grain boundaries.

## 1. Introduction

Increasing the steam temperature of modern coal-fired power plants is the best approach to improve the efficiency of fossil fuel and reduce pollutants. In recent years, more and more ultra-supercritical (USC) and supercritical (SC) power plants have been built worldwide. For example, as of now, almost 100 ultra-supercritical and supercritical power plants have been built in China. However, proper materials for superheaters and reheaters, which are the hottest in the whole power plant, are still challenged, especially for the use of coarse-grain and fine-grain heat-resistant steel. One of the most important factors for material selection is the oxidation resistance at elevated temperatures in steam [1,2,3,4,5]. 

There are many papers describing the steam oxidation of heat-resistant steel used in USC power plants [6,7,8,9,10,11,12,13,14,15,16]. It is well known that Cr is the indispensable element in heat-resistant steel, with excellent strength and corrosion resistance. A stable chromia layer forms on the surface of Cr-containing steels and alloys at elevated temperatures to provide protection against severe environments [17,18,19,20]. Viswanathan et al. [1] concluded that steam oxidation of heat-resistant steel was influenced by the heat flux, environment, steam parameter, and alloy compositions. Surface pretreatments and sample geometry also affected the oxidation resistance of steels, such as sandblasting and pre-oxidation treatment [1,16,21]. Fry and Piedra [22] discussed the effect of specimen geometry, steam pressure, and dissolved oxygen of water on the oxidation behavior of heat-resistant steel. However, the effect of grain refinement on the steam oxidation of austenitic heat-resistant steels was not fully understood. Peng et al. [23] investigated the effect of grain refinement on the resistance of 304 stainless steel in wet air. They found that abundant grain boundaries greatly increased the outer diffusion of Cr ions to guarantee the growth of Cr-rich oxide scales. Perez [24] studied the influence of grain size on the oxidation behavior of PM2000 in the air. He confirmed that grain boundaries acted as rapid pathways for the diffusion of the aluminum ion. Nevertheless, Yan et al. [25] investigated the steam oxidation of austenitic stainless. Their results showed that the breakaway oxidation of fine-grain R304H and TP347HFG was observed, which was not the case for coarse-grain W304H steel. They explained that the grain boundaries promoted the Fe outward diffusion and faster growth of interfacial voids. Research attention was attracted by the contrary results of the effect of grain size on the oxidation resistance of heat-resistant steel.

The aim of this paper is to investigate the steamoxidation behavior of austenitic heat-resistant steels TP347HFG and TP347H. 

## 2. Experimental System and Methods

**Figure 1:**
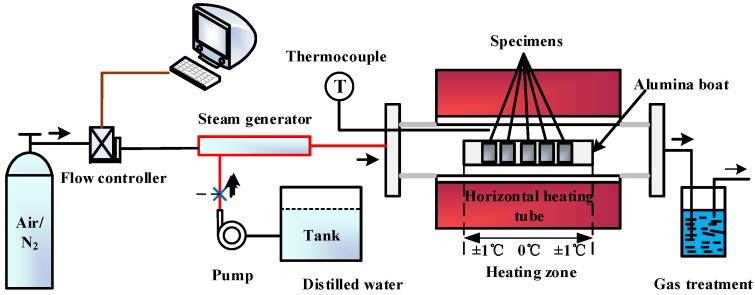
Schematic diagram of the steam oxidation system. Adapted from [26], with permission from © 2015 Spring Nature.

The steam oxidation test setup is shown in Figure 1. The system consisted of a gas flow controller, a steam generator, and a horizontal tube furnace with an alumina tube. An internal thermocouple was used to measure and control the temperature of the test samples. In the steam generator, water was continuously introduced at the rate of 6 mL/h, into a separately heated stainless-steel vessel to generate a continuous flow of steam, with a rate of 134.4 mL/h for the oxidation experiments. Specimens were placed perpendicular to the alumina boat surface so that they were in a constant temperature zone and did not affect steam flow to other samples.

**Figure 2:**
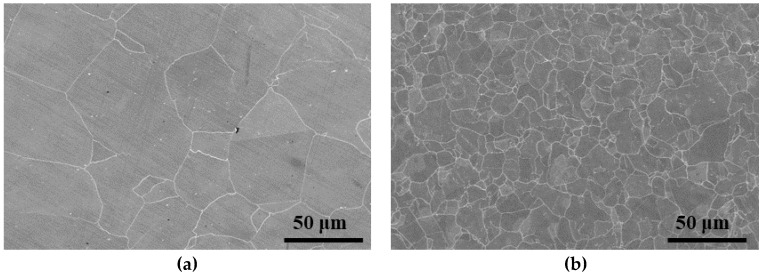
Microstructures of TP347H and TP347HFG. (**a**) TP347H; (**b**) TP347HFG.

The compositions of steels TP347H and TP347HFG are listed in Table 1, which are used for reheaters and superheaters in modern USC boilers [1,15,17]. Steels TP347H and TP347HFG were provided by Shanghai Boiler Works Ltd. (Shanghai, China). Coarse-grained TP347H and fine-grained TP347HFG were normalized at 1150 °C for 13 min (water cooled) and 1180 °C for 7 min (water cooled), respectively. Figure 2 shows the microstructures of TP347H and TP347HFG. The average grain sizes of TP347H and TP347HFG were 51.3 μm and 14.5 μm, respectively. Samples with a size of 15 mm × 15 mm × 2.8 mm were ground by 120#, 400#, and 1000# SiC papers and ultrasonically cleaned in ethanol for 5 min. Specimens were weighed using an electronic balance with an accuracy of 10^−4^ g before and after the experiment to obtain the mass change during the oxidation test. Mass gain curves were obtained using five samples and each one was removed from the furnace for different times. After the oxidation test, the oxide scale formed on the sample surface was characterized by XRD (X’Pert PRO, PANalytical, Eindhoven, The Netherlands) using Cu K-alpha radiation. The scanning range was 20°–80°. The voltage and the current used were 25 kV and 40 mA, respectively. 

For another set of test samples, the cross-sectional morphology was observed using a scanning electron microscope (SEM, SU3500, Hitachi, Tokyo, Japan) with energy dispersive spectroscopy (EDS, Oxford, Oxford Instruments, London, UK). X-ray photoelectron spectroscopy (XPS, Thermo Scientific K-Alpha, Thermo Fisher Scientific, Waltham, MA, USA) was used to characterize the main oxidation products.

## 3. Results and Discussion

**Figure 3:**
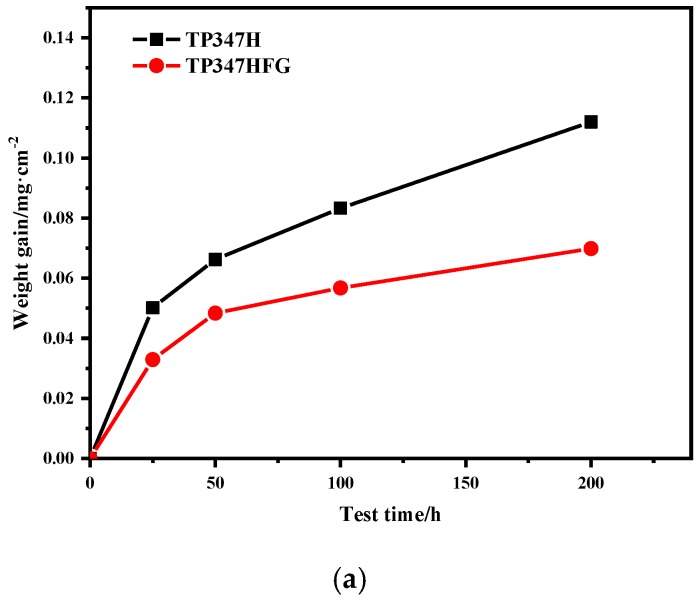

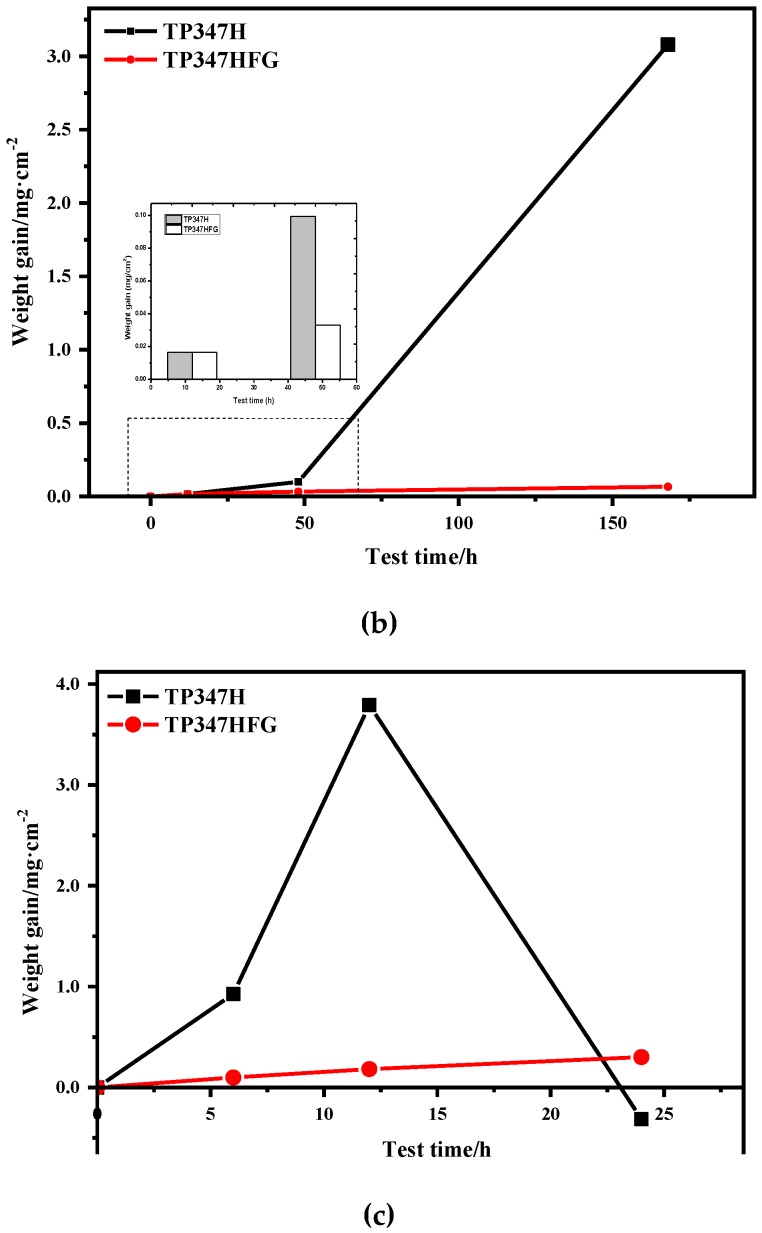
Weight gain curves ofTP347H and TP347HFG in a steam environment. (**a**) 650 °C; (**b**) 700 °C; (**c**) 800 °C.

Figure 3 shows the weight gain curves of TP347H and TP347HFG at 650 °C, 700 °C, and 800 °C in a steam environment. Weight gains of TP347H were much higher than that of TP347HFG at all experimental temperatures. Weight gain gaps between TP347H and TP347HFG at 700 °C and 800 °C were much bigger, as shown in Figure 3b,c. The oxidation kinetics of TP347HFG approximately followed a parabolic law at all temperatures, while the oxidation kinetics of TP347H only followed a parabolic law at 650 °C. Weight gain of TP347H increased sharply due to the probable failure of the protective oxide scale. Higher temperatures promoted the failure of the protective oxide scale, leading to the breakaway oxidation of TP347H. The oxidation constants of steel at 650 °C, 700 °C, and 800 °C were calculated by fitting weight gain curves, as listed in Table 2.

**Figure 4:**
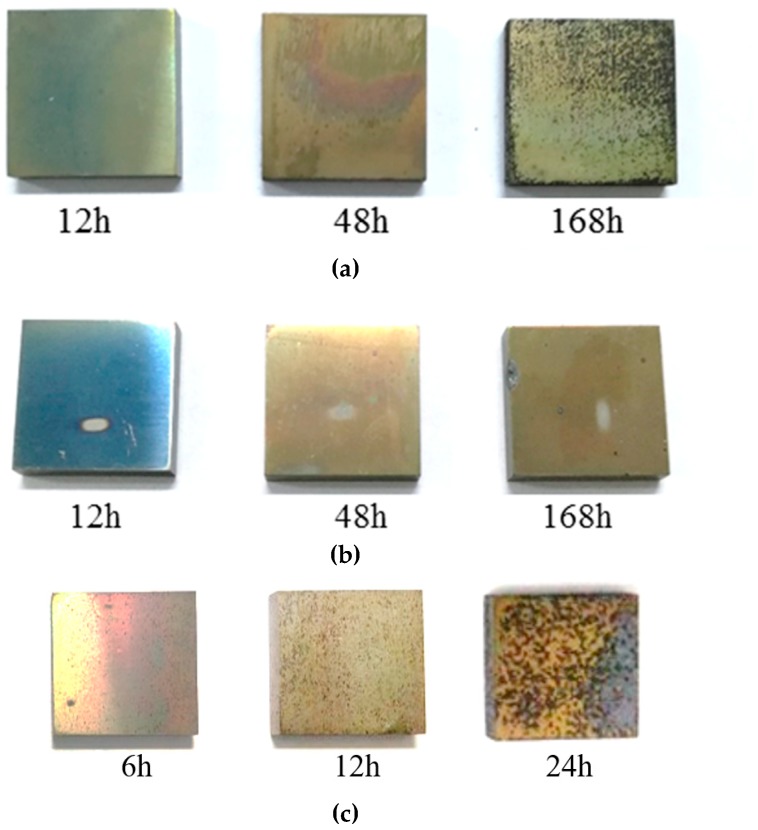
Macroscopic morphologies of austenitic heat-resistant steels at 700 °C and 800 °C. (**a**) TP347H at 700 °C; (**b**) TP347HFG at 700 °C; (**c**) TP347H at 800 °C.

Figure 4 displays the macroscopic morphologies of TP347H and TP347HFG at 700 °C after exposure to the steam for 12 h, 48 h, and 168 h and at 800 °C for 24 h. No exfoliation of oxide scales was found in any sample at 700 °C in Figure 4a,b, but some oxide scales spalled at 800 °C, as shown in Figure 4c. With a longer experiment time, the color of TP347HFG was converted from blue to faint yellow, which agreed with the published literature [27]. Note that numerous black dots were observed at the surface of TP347H after 168h, which was responsible for the higher weight gain of TP347H in Figure 3b.

**Figure 5:**
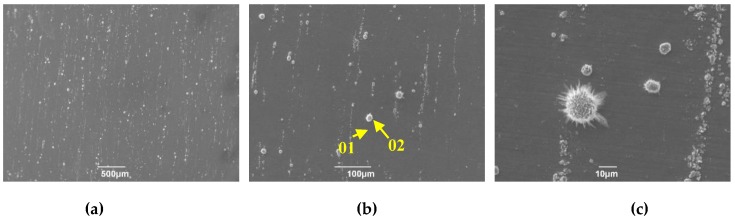

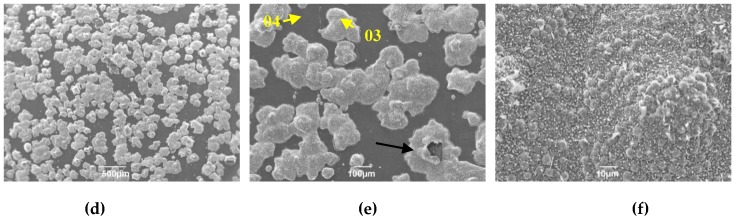
Surface morphology of TP347H and TP347HFG at 700 °C after 168 h. (**a**) TP347HFG; (**b**) TP347HFG; (**c**) TP347HFG; (**d**) TP347H; (**e**) TP347H; (**f**) TP347H.

Figure 5 displays surface microscopic morphology of TP347H and TP347HFG at 700 °C after exposure to the steam for 168 h. Some independent oxide particles were observed on the plain surface of TP347HFG, as shown in Figure 5a,b. The surface of TP347H was covered by some big, island-like oxides. Some island-like oxides were connected together, as shown in Figure 5d. At a larger magnification, the breakaway of the oxide scale was detected on the surface of TP347H, as shown in Figure 5e. This phenomenon was closely related to the release of hydrogen in oxide scales [2], which caused the exfoliation of the outer oxides scale on TP347H. In Figure 5f, we found many spherical oxides at the island-like oxidation products, which is in accord with the occurrence of the hydrogen release reaction. The hydrogen was originally from the reaction between the metal or metal oxide and the steam [28]. 

**Figure 6:**
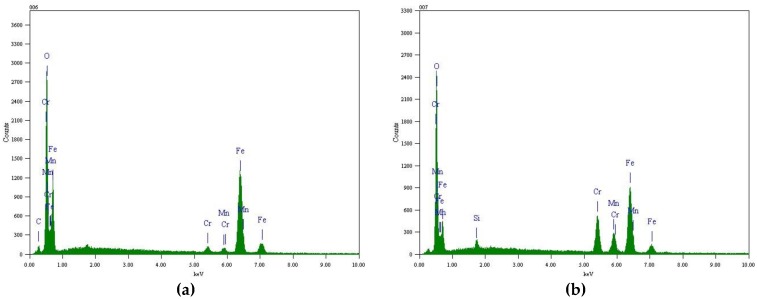
EDS analysis of the oxides formed on TP347H at 700 °C after 168 h. (**a**) No.03 in Figure 5; (**b**) No.04 in Figure 5.

**Figure 7:**
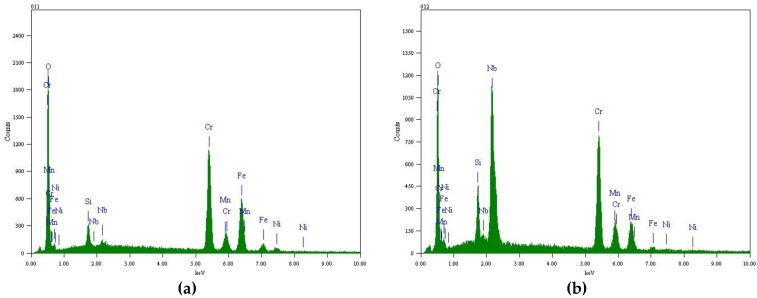
EDS analysis of the oxides formed on TP347HFG at 700 °C after 168 h. (**a**) No.01 in Figure 5; (**b**) No.02 in Figure 5.

Figure 6 and Figure 7 show EDS analysis of the surface oxides formed on TP347H and TP347HFG at 700 °C. The island-like products at the surface of TP347H were mainly composed of Fe oxides, as shown in Figure 6a. The plain surface between these oxide islands was covered by Cr-Mn-Fe oxides, of which Cr-rich oxides dominated, as shown in Figure 6b. This Cr-Mn-Fe oxide scale improved the oxidation resistance of heat-resistant steel in steam at higher temperatures. The independent oxide particles labeled by the point 01 in Figure 5b on TP347HFG were mainly Nb-rich oxides, which were surrounded by Cr-rich oxides, as shown in Figure 6. Moreover, element Mn was detected on the surface of TP347H and TP347HFG, as listed in Table 3.

**Figure 8:**
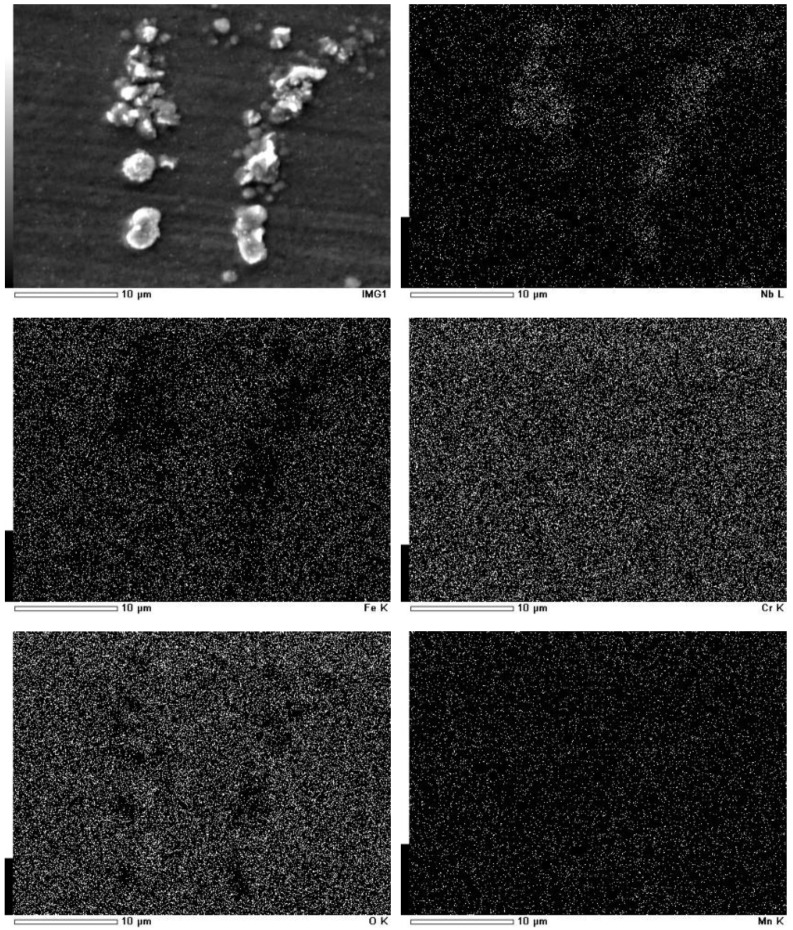
Element distribution of surface oxides on TP347HFG at 700 °C after 168 h.

**Figure 9:**
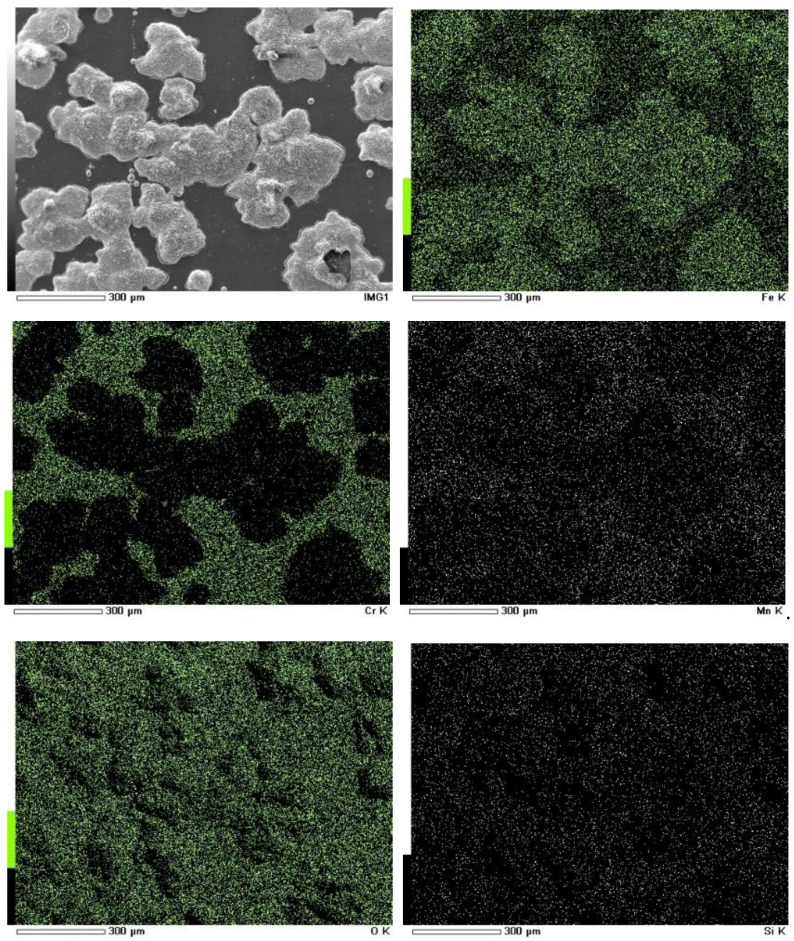
Element distribution of surface oxides on TP347H at 700 °C after 168 h.

Figure 8 and Figure 9 show the elemental distribution of surface oxides of TP347H and TP347HFG at 700 °C after 168 h. Some extrusive Nb oxides on TP347HFG were covered by Fe-Cr oxides. For TP347H, the oxide scales were mainly composed of island-like Fe oxides and Cr-Mn oxides at other positions. Associated with morphology variation of TP347H in Figure 4a and weight gain in Figure 2, we confirmed that this steam oxidation was the breakaway oxidation.

**Figure 10:**
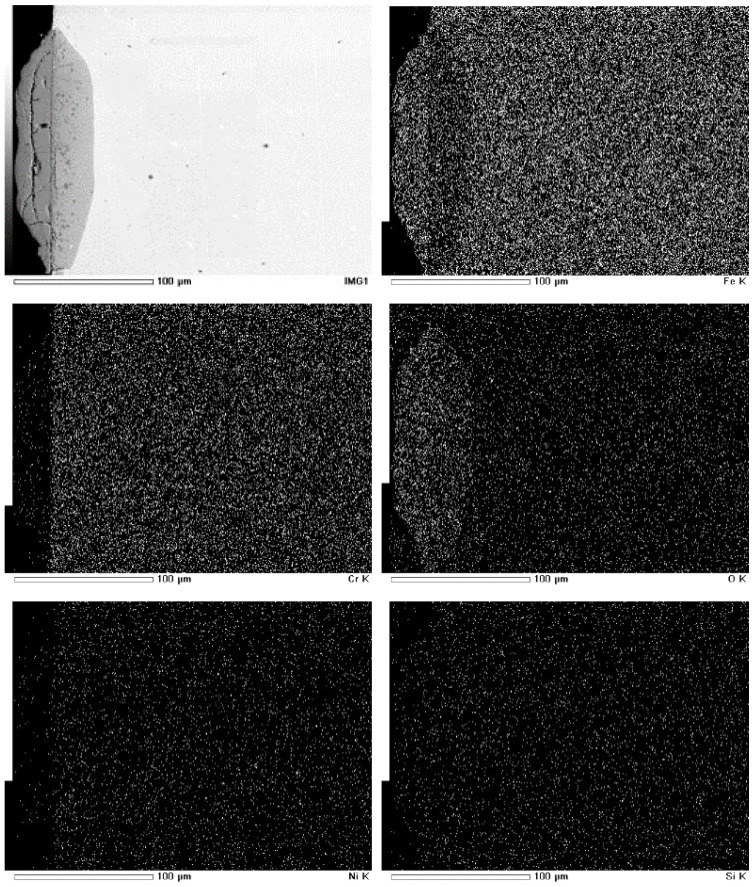
Cross-sectional morphology and elemental mapping of TP347H at 700 °C.

**Figure 11:**
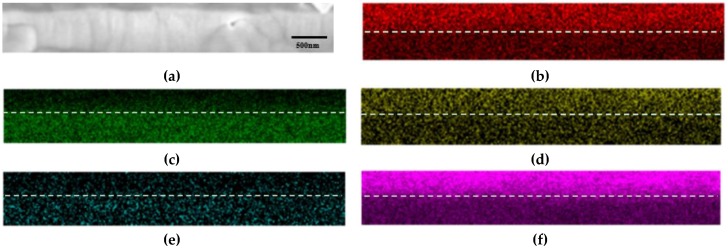
Cross-sectional mapping of TP347HFG at 700 °C. (**a**) SEM figure; (**b**) Cr; (**c**) Fe; (**d**) Mn; (**e**) Ni; (**f**) O.

Figure 10 and Figure 11 display the cross-sectional morphology and elemental mapping of TP347H and TP347HFG after 168 h at 700 °C. The oxide scales on TP347H were composed of an outer layer of Fe-oxides and an inner layer of Fe-Cr oxides. Some cracks and pores were detected at the outer layer on TP347H, which may have caused the exfoliation of the outer layer. Because the oxide scales on TP347HFG were very thin, the focused ion beam technique was used to obtain the cross-sectional morphology of TP347HFG. The thin and protective oxide scales were observed on TP347HFG, which were mainly Cr oxides and Mn-Cr oxides, as shown in Figure 11. This result agreed with the weight gain results in Figure 3, because thin Cr-rich oxide scales on TP347HFG provided good resistance against the steam environment.

**Figure 12:**
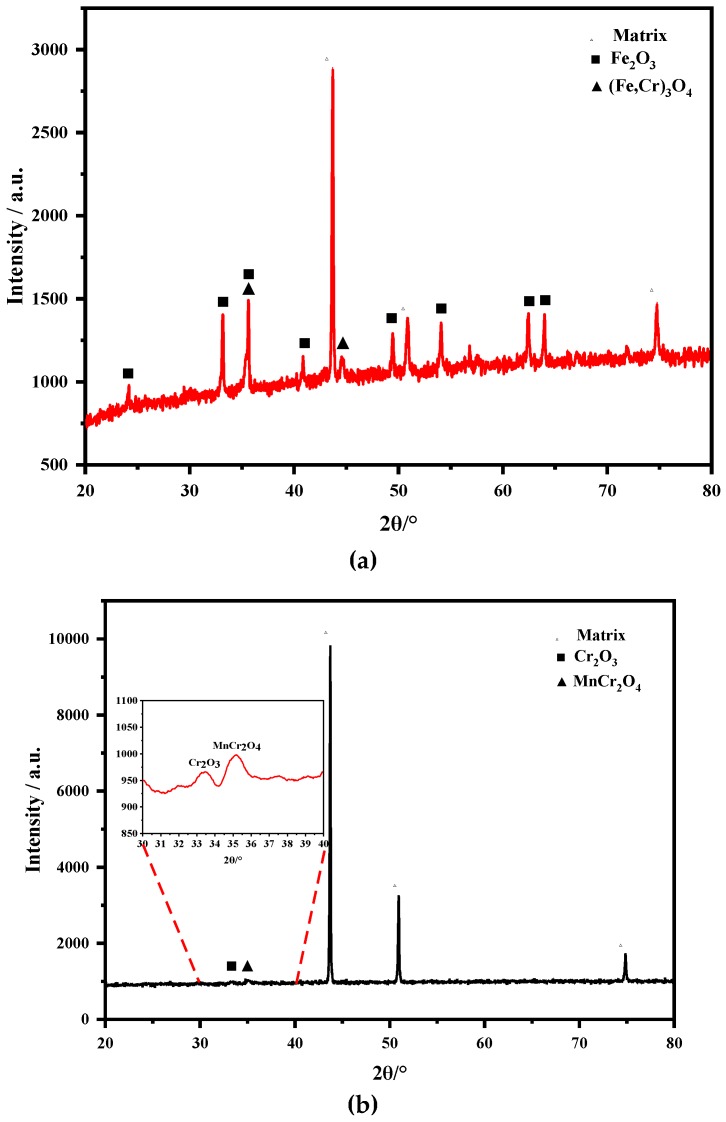
XRD results of TP347H and TP347HFG after 168 h at 700 °C. (**a**) TP347H; (**b**) TP347HFG.

Figure 12 shows the XRD results of the oxide scales on TP347H and TP347HFG at 700 °C. The oxide scales on TP347H were composed of Fe_2_O_3_ and (Fe,Cr)_3_O_4_ in Figure 12a, while oxide scales on TP347HFG consisted of Cr_2_O_3_ and MnCr_2_O_4_ in Figure 12b. From the XRD results and the EDS mapping results in Figure 10 and Figure 11, it was confirmed that the outer and inner oxide layers of TP347H were Fe_2_O_3_ and (Fe,Cr)_3_O_4_ from the steam/oxide scale interface to the substrate of TP347H.

**Figure 13:**
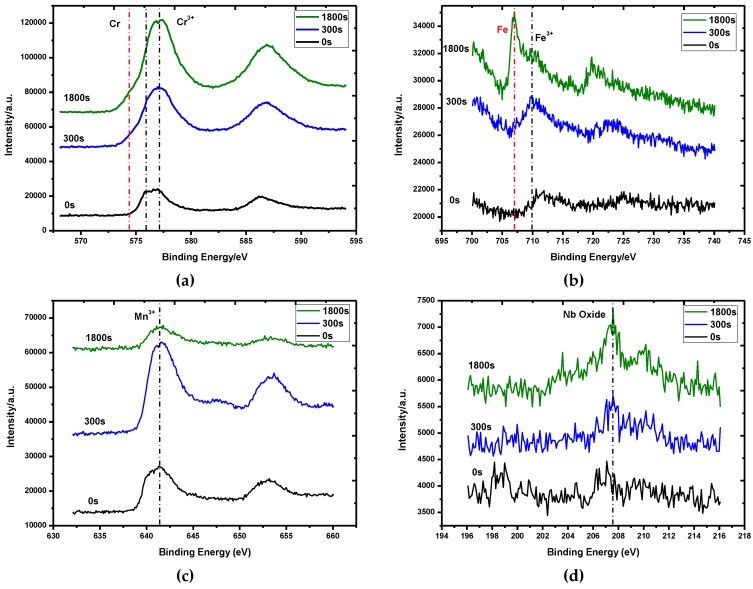
XPS results of the oxide scale of TP347HFG at 700 °C after 168 h. (**a**) Cr 2p_3/2_; (**b**) Fe 2p_3/2_; (**c**) Mn 2p_3/2_; (**d**) Nb 3d.

Figure 13 shows XPS results of the oxide scales of TP347HFG at 700 °C after 168 h. The main elements were Cr, Fe, Mn, and O. Figure 13 shows the XPS high-energy resolution spectra of Fe 2p, Cr 2p, Mn 2p, and Nb 3d. Depth profiling by argon sputtering was obtained for different times to get the chemical state of elements at different sputtered depths. Depth-resolved Fe 2p_3/2_ spectra shown in Figure 13b positioned the binding energy ranging from 711 eV to 709.9 eV, which corresponded to Fe-oxides. After 0.5 h sputtering, metallic Fe peak at 706.9 eV indicated the oxide scale/the substrate interface. Cr 2p_3/2_ spectra were located at the binding energy values of 576.7 eV and 575.7 eV. The binding energy peak at 576.7 eV was thought to be Cr_2_O_3_ and the lower one was (Fe,Cr)_3_O_4_. Despite the low content of Mn in TP347HFG, a high degree of segregation and its oxides were observed. The binding energy value of 641.6 eV for Mn 2p_3/2_ spectra in Figure 13c corresponded to Mn-oxides, including MnCr_2_O_4_ and MnO. The Nb 3d structure in Figure 13d showed a peak up to the sputtering time of 0.5 h at 207 eV, which indicated that Nb was oxidized to Nb_2_O_5_. The XPS results indicated that the oxide scales were mainly composed of MnCr_2_O_4_ and Cr_2_O_3_ from the gas/solid interface to the substrate.

## 4. Discussion

The weight gain of TP347H increased sharply from 650–800 °C after a slow weight gain, while the weight gain of TP347HFG followed a parabolic law. Breakaway oxidation was observed on the TP347H at 700 °C and 800 °C, which was different from Yan’s result [25], which was that breakaway oxidation occurred on fine-grain steel at 620 °C. From the results of weight gain and microstructures of austenitic heat-resistant steel TP347H and TP347HFG, it was concluded that the initial protective Cr-rich oxide scale formed on TP347H failed because of the coarse grain of TP347H. The initial protective Cr-rich oxide scale was mainly composed of lamellated MnCr_2_O_4_ and Cr_2_O_3_ layers, which are validated by the XRD results in Figure 12b, the EDS results in Figure 11, and the XPS results in Figure 13. After a longer experiment time, the breakaway oxidation was confirmed by the greater weight gain seen in Figure 2 and the island-like Fe oxides seen in Figure 9 and Figure 10. Many Fe_2_O_3_ oxides were characterized at the failure position of the original Cr-rich oxide scales, as shown in Figure 3b and Figure 9. From the viewpoint of oxidation kinetics and oxidation nature, the breakaway oxidation was decided by the ion diffusion and the evaporation of chromia in a steam environment at high temperatures [23,29,30]. In this study, the different oxidation behaviors of TP347H and TP347HFG were caused by the different grain sizes in Figure 2. The breakaway oxidation behavior was found on TP347H, as shown in Figure 3 and Figure 5f. For the ion diffusion during the oxidation process, the outward diffusion rates of metallic ions via the grain boundary (GB) are at least ten times that via the grain bulk, so grain boundaries play a significant role in the ion diffusion [28]. As we know, the diffusion rate is usually expressed by the effective diffusion coefficient *D*_eff_, which is described in the following Equations (1) and (2).

(1) $$D_{\mathrm{eff}}={fD}_{b}+(1-f)D_{l}$$

(2) f=qw/d

where *D*_l_ is the diffusion coefficient via the grain, *D*_b_ is the diffusion coefficient at the grain boundary, *q* is the value decided by the grain shape, *w* is the width of the grain boundary, and *d* is the grain size. *D*_eff_ values of Cr and Mn are larger when the grain size *d* is smaller, so the *D*_eff_ values of Cr and Mn on TP347HFG are much larger than those on TP347H, resulting in the sufficient outward diffusion of Mn and Cr ions during the oxidation, leading to the stable oxides formed on TP347HFG, as shown in Figure 3. The rapid diffusion of metallic ions in TP347HFG provides sufficient Cr and Mn for the formation of Cr-rich oxide scales in Figure 8. For TP347H, nodule Fe oxide scales were attributed to the coarse grain, which cannot provide sufficient Cr and Mn ions. There are two reasons explaining the effect of Mn and Cr on the oxidation resistance of TP347H and TP347HFG.

First of all, the outward diffusion of Mn and Cr is much faster than that of other metallic ions at higher temperatures [31]. Secondly, due to the lower free energy of the formation of MnCr_2_O_4_, which can be calculated by the following equations, duplex oxide layers were developed at the surface of austenitic steels containing Mn at the early oxidation stage, as shown in the XRD and XPS results in Figure 12 and Figure 13, respectively. The MnCr_2_O_4_ oxide scale other than Cr_2_O_3_ could effectively hinder the evaporation of CrO_2_(OH)_2_ [32,33], improving the oxidation resistance of TP347HFG.

(3) $$2\mathrm{Mn}+O_{2(g)}=2{\mathrm{MnO}}_{\left(s\right)},\;\Delta G_{T}^{\theta }=-856.3+0.1825T$$

(4) $$4/3\mathrm{Cr}+O_{2(g)}=2/3\;{\mathrm{Cr}}_{2}O_{3(s)},\;\Delta G_{T}^{\theta }=-753.12+0.1826T$$

(5) $$\mathrm{MnO}+{\mathrm{Cr}}_{2}O_{3(g)}={\mathrm{MnCr}}_{2}O_{4\;(s)},\;\Delta G_{T}^{\theta }=-1469.2+0.2798T$$

where $\Delta G_{T}^{\theta }$ is the change of free energy and *T* is the reaction temperature.

On the other hand, considering the dissolved oxygen content in distilled water of 9.08 mg/L at 25 °C, the evaporation of chromia in a steam environment occurred at 650–800 °C in Equations (3) and (4), which has been confirmed by Asteman and Young [34,35,36,37,38]. In USC power plants, complex chromium oxyhydroxides were also detected in the steam, which proved the experimental data [35,36].

2Cr_2_O_3_ + 3O_2_ = 4CrO_3_ (g) (6)

2Cr_2_O_3_ + 4H_2_O + 3O_2_ = 4CrO_2_(OH)_2_ (7)

The calculated values of ***P***CrO_2_(OH)_2_ at 650–800 °C in a steam environment are listed in Table 4 according to the curve in literature [38]. The ***P***CrO_2_(OH)_2_ at 800 °C was ten times than that at 700 °C and 650 °C, leading to the abnormal weight gain after 100 h at 700 °C and 24 h at 800 °C (Figure 3b). The values of ***P***CrO_2_(OH)_2_ at 800 °C indicate quicker consumption of Cr_2_O_3_. Due to the quicker consumption of Cr_2_O_3_ and the slower diffusion of beneficial Cr and Mn ions on TP347H, the incubation period for breakaway oxidation of Cr-rich oxide scales on TP347H at high temperatures was much shorter, as shown in Figure 3. 

## 5. Conclusions

(1) The weight gain of TP347HFG at 650–800 °C was much lower than that of TP347H. The oxidation kinetics of TP347HFG nearly followed a parabolic law. Breakaway oxidation behaviors were observed on TP347H at 700 °C and 800 °C.
(2) Duplex oxide scales formed on TP347HFG were composed of MnCr_2_O_4_ and Cr_2_O_3_ from the steam/oxide scales interface to the substrate. The oxide scales on TP347H consisted of Fe_2_O_3_ nodules and Fe-Cr oxide scale. This result was decided by the grain size, which provides quick outward diffusion of metallic ions at the grain boundaries.
(3) The thin and protective Cr-rich oxides were replaced by Fe_2_O_3_ nodules, which was attributed to insufficient outward migration of metallic ions, including Cr and Mn at the subsurface of coarse-grain TP347H. The fine grain of TP347HFG improved its oxidation resistance against the steam environment.
(4) Quick evaporation of the Cr_2_O_3_ oxide scale and the slower outward diffusion of metallic ions at higher temperatures led to the shorter incubation period for breakaway oxidation of the Cr-rich oxide scales on TP347H.

## Figures and Tables

**Table 1:** Compositions of steels TP347H and TP347HFG (wt.%).

Materials	C	Mn	Si	S	P	Cr	V	Ni	Cu	Nb
TP347H	0.07	1.19	0.39	0.002	0.015	18.39	<0.3	10.1	<0.03	0.73
TP347HFG	0.08	0.75	0.48	0.002	0.020	18.31	<0.1	10.7	0.05	0.73

**Table 2:** Oxidation constants of TP347H and TP347HFG.

Materials	650 °C	700 °C	800 °C
TP347H	Δm = 0.00796*t*^0.5^	-	-
TP347HFG	Δm = 0.00526*t*^0.5^	Δm = 0.00537*t*^0.5^	Δm = 0.00595*t*^0.5^

**Table 3:** EDS analysis of the oxides formed on TP347HFG and TP347H (wt.%).

Content	Cr	Fe	O	Mn
No.01	34.53	22.68	33.73	4.45
No.03	25.82	35.25	23.88	3.53

**Table 4:** ***P***CrO_2_(OH)_2_ in steam at 650–800 °C.

Temperature (°C)	650	700	800
*P*CrO_2_(OH)_2_ (atm)	1.4 × 10^−11^	4.0 × 10^−11^	4.0 × 10^−10^
